# Kampo Medicines Modulate Angiogenic, Antioxidant, and Inflammatory Pathways in Human Preclinical Models: Implications for Preeclampsia

**DOI:** 10.3390/antiox15070877

**Published:** 2026-07-14

**Authors:** Natalie K. Binder, Kenji Onda, Sally Beard, Kei Uchiyama, Chika Ohi, Natasha de Alwis, Lydia Baird, Tu’uhevaha J. Kaitu’u-Lino, Toshihiko Hirano, Haruki Yamada, Toshihiro Sakurai, Natalie J. Hannan

**Affiliations:** 1 Therapeutic Discovery and Vascular Function in Pregnancy Group, University of Melbourne and Mercy Hospital for Women, Heidelberg 3084, Australia; 2 Mercy Perinatal, Mercy Hospital for Women, Heidelberg 3084, Australia; 3 Department of Clinical Pharmacology, Tokyo University of Pharmacy and Life Sciences, Tokyo 192-0392, Japan; 4 The Walter and Eliza Hall Institute of Medical Research, Parkville 3052, Australia; 5 Translational Obstetrics Group, University of Melbourne and Mercy Hospital for Women, Heidelberg 3084, Australia; 6 Department of Kampo Medicine, Tokyo University of Pharmacy and Life Sciences, Tokyo 192-0392, Japan; 7 Department of Pharmaceutical Sciences, Ohu University, Koriyama 963-8611, Japan

**Keywords:** endothelial dysfunction, herbal medicine, angiogenesis, oxidative stress, placenta

## Abstract

Preeclampsia is a serious pregnancy complication characterised by maternal vascular dysfunction, placental dysfunction, and organ injury, with no effective treatment currently available. Kampo, a system of Japanese traditional medicine comprising standardised herbal formulations, could target pathophysiological pathways driving preeclampsia. We evaluated the effects of select Kampo formulations on markers of preeclampsia using primary human trophoblasts, placental explants, human umbilical vein endothelial cells (HUVECs), and uterine microvascular endothelial cells (UtMVECs). Twelve formulations were initially screened in HUVECs, and six formulations advanced for further study. TNFα was used to induce endothelial dysfunction, and angiogenic, antioxidant, inflammatory, and vascular dysfunction markers were assessed. Overall, Kampo formulations had minimal effect on sFlt-1 expression and only modest effects on sFlt-1 secretion by primary human trophoblast. In contrast, several formulations consistently increased placental growth factor (PlGF) expression and secretion, upregulated HMOX1 in trophoblasts, and enhanced PlGF secretion from placental explants. In endothelial cells, Kampo treatment partially reversed TNFα-induced dysfunction, demonstrated by reduced VCAM1 expression, and additional endothelial cell type-dependent effects on ET-1 and inflammatory pathways. These findings indicate that selected Kampo formulations modulate key pathways involved in the pathophysiology underpinning preeclampsia and warrant further investigation as potential therapeutic candidates.

## 1. Introduction

Preeclampsia is a serious hypertensive disorder of pregnancy characterised by new-onset hypertension and proteinuria or end-organ dysfunction after 20 weeks’ gestation [1]. Affecting 5–8% of pregnancies worldwide, preeclampsia is a leading cause of maternal and perinatal morbidity and mortality [2,3,4]. The pathogenesis of preeclampsia involves abnormal placentation early in pregnancy, leading to placental hypoxia/oxidative stress and the release of anti-angiogenic factors into the maternal circulation [5,6,7]. This promotes endothelial dysfunction and a systemic inflammatory response, resulting in the clinical manifestations of the disease [8,9].

Despite its substantial impact and disease burden [10], there are currently no effective treatments for preeclampsia once established, other than delivery of the placenta, which often necessitates premature birth [11]. This therapeutic gap underscores an urgent need to identify novel interventions that can target and mitigate the key pathological features of preeclampsia [12], including placental insufficiency and oxidative stress, systemic inflammation, vascular and immune dysfunction, angiogenic/anti-angiogenic factor imbalance, and neurological symptoms.

Traditional Japanese herbal medicine, known as Kampo, has been practised for centuries and is increasingly recognised for its potential therapeutic applications in modern medicine [13,14,15,16,17,18]. Kampo formulations are composed of carefully balanced combinations of multiple natural herb extracts standardised into one formulation, with each herbal extract formulation tailored to specific physiological conditions [19], especially with respect to oxidative stress. Their mechanisms of action often include anti-inflammatory, antioxidant, and immunomodulatory effects, which could make them promising candidates for the treatment of preeclampsia. Additionally, Kampo medicines are widely used in Japan and have established safety profiles, particularly in perinatal care [20,21].

Despite the widespread clinical use of Kampo formulations for a range of conditions, their potential to target mechanisms underlying preeclampsia remains largely unexplored. Here, we investigated twelve Kampo formulations: Otsujito, Goreisan, Tokishakuyakusan, Keishibukuryogan, Rikkunshito, Shichimotsukokato, Shakuyakukanzoto, Shimotsuto, Ryutanshakanto, Tokiinshi, Tokito, and Saireito, to determine their capacity to modulate key pathophysiological features of preeclampsia.

The specific Kampo formulations were carefully selected either due to their prior reported use in pregnancy-related conditions in Japan, and/or their specific benefits in other conditions, where examples of recommended use could extend to associated treatment of the pathophysiology driving preeclampsia.

Otsujito is traditionally used for managing constipation and haemorrhage in pregnancy. Goreisan is recommended for pregnancy-associated oedema and amniotic fluid reduction, contributing to water balance regulation. Tokishakuyakusan functions as an *anthaiyaku* (formulation for pregnancy maintenance) and has been used to manage pregnancy-induced hypertension, as well as for the prevention of miscarriage and preterm birth. Keishibukuryogan is commonly prescribed to alleviate pregnancy-related varicose veins/vascular issues. Rikkunshito is indicated for hyperemesis gravidarum, abdominal pain, and anaemia. Shichimotsukokato is also employed in cases of pregnancy-induced hypertension. Shakuyakukanzoto is utilised for managing muscle cramps and is suggested for the prevention of preterm birth. Shimotsuto is recommended for conditions such as varicose veins and iron deficiency/anaemia during pregnancy. Ryutanshakanto possesses anti-inflammatory properties and is occasionally used to address urinary tract inflammatory oxidative stress issues.

Tokiinshi is prescribed for dermatological issues, including skin pruritus, chronic eczema, and atopic dermatitis. Tokito is used for cold-induced chest or abdominal pain and irregular menstruation; however, its application in pregnancy-related conditions is less documented. We included Tokito in this study because other toki (*Angelica acutiloba*) containing formulations, including Otsujito, Tokishakuyakusan, Shichimotsukokato, Shimotsuto, Ryutanshakanto, and Tokiinshi, are frequently utilised in pregnancy-related conditions. Saireito is recommended to prevent pregnancy-induced hypertension, the prevention of miscarriage, recurrent pregnancy loss, as well as alleviation of oedema.

We set out to investigate the effects of these select Kampo formulations in models of preeclampsia. Specifically, we aimed to evaluate the ability of these Kampo formulations to balance altered expression of key angiogenic factors, boost antioxidant cytoprotective enzymes, and markers of endothelial dysfunction in primary human models (placental tissue explants, cytotrophoblast, and endothelial cells). Our intention was to identify whether specific Kampo formulations had the potential to alleviate the key pathophysiological features of preeclampsia. These findings would provide a foundation for Kampo medicines and their careful future evaluation in clinical settings. To our knowledge, this is the first study to systematically evaluate the effects of Kampo medicines on multiple pathophysiological pathways relevant to preeclampsia using primary human cells and placental tissues.

## 2. Materials and Methods

### 2.1. Tissue Collection

Ethical approval for this study was obtained from the Mercy Health Human Research Ethics Committee (R11/34). Women presenting to the Mercy Hospital for Women, Heidelberg, Australia, gave informed written consent for tissue collection. Placentas and umbilical cords were collected from normal term pregnancies (between 38 + 3 and 40 + 5 weeks of gestation) at elective caesarean section. Samples were collected within 30 min of delivery and washed in cold phosphate-buffered saline (PBS).

### 2.2. Primary Human Cytotrophoblast Isolation

Human cytotrophoblasts were isolated from three to five individual placentas per experiment, as previously described [22]. Primary cytotrophoblasts were cultured in DMEM GlutaMAX (Life Technologies; Carlsbad, CA, USA) containing 10% fetal calf serum (HyClone, Logan, UT, USA) and 1% antibiotic–antimycotic (Life Technologies, Carlsbad, CA, USA) on fibronectin-coated wells (10 mg/mL; BD Biosciences; San Jose, CA, USA). Cells were plated and allowed to attach for over 12–18 h before washing with dPBS (Life Technologies) to remove cell debris. Cells were cultured under 8% O_2_, 5% CO_2_ at 37 °C.

### 2.3. Placental Explant Culture

Placental explants were dissected from three to five individual placentas per experiment. Small pieces of villous tissue were cut from multiple sites of the placenta, with removal of the maternal and fetal surfaces. These were thoroughly washed with PBS, then dissected into small fragments of 1–2 mm size, and three pieces were put into each well of a 24-well plate. Explants were allowed to equilibrate at 37 °C for 12–18 h under 8% O_2_, 5% CO_2_ in DMEM GlutaMAX containing 10% fetal calf serum and 1% antibiotic–antimycotic.

### 2.4. Primary Human Endothelial Cell Isolation

Primary human umbilical vein endothelial cells (HUVECs) were commercially purchased from Takara Bio (Shiga, Japan) or isolated from three to five individual umbilical cords per experiment, as previously described [23]. Briefly, the umbilical cord vein was cannulated and flushed with PBS to wash out blood cells. Next, 10 mL of collagenase (1 mg/mL, Life Technologies) was infused into the cord and incubated at 37 °C for 10 min. The dissociated HUVECs were recovered by pelleting and resuspension, followed by culture in M199 media (Life Technologies) containing 20% fetal calf serum, 1% antibiotic–antimycotic, 1% endothelial cell growth factor (Sigma; St. Louis, MO, USA), and 1% heparin (Sigma). Cells were used between passages 2 and 4 and cultured at 37 °C in 20% O_2_ and 5% CO_2_.

Primary human uterine microvascular endothelial cells (UtMVECs, Lonza, Basel, Switzerland) were cultured in Clonetics^TM^ Endothelial Cell Growth Medium-2 (Lonza; Basel, Switzerland). Cells were used between passages 14 and 16 and cultured at 37 °C in 20% O_2_ and 5% CO_2_.

### 2.5. Kampo In Vitro Experiments

Commercially manufactured spray-dried Kampo extract granules were provided by Dr Onda and Dr Yamada, manufactured by Tsumura & Co. (Tokyo, Japan). Standardised extract formulations were dissolved in sterile PBS for the preparation of stock formulations. Each Kampo stock formulation (Tsumura, Tokyo, Japan) was then prepared to 10 mg/mL in culture medium, vortexed at room temperature, and centrifuged before sterile filtration (0.22 µm; Polyethersulfone (PES) membrane) of the resulting supernatants. Cell viability assays (CellTiter 96 AQueous Non-Radioactive Cell Proliferation Assay, Promega; Madison, WI, USA) according to the manufacturer’s instructions to select non-cytotoxic concentrations for experimentation.

For initial screening, commercially sourced HUVECs were treated with 12 Kampo formulations selected for their documented use in pregnancy-related conditions in Japan at two concentration dose ranges to define the concentration-effect relationship: Otsujito (0.625 and 1.25 mg/mL), Goreisan (5 and 10 mg/mL), Tokishakuyakusan (2.5 and 5 mg/mL), Keishibukuryogan (1.25 and 2.5 mg/mL), Rikkunshito (5 and 10 mg/mL), Shichimotsukokato (2.5 and 5 mg/mL), Shakuyakukanzoto (1.25 and 2.5 mg/mL), Shimotsuto (2.5 and 5 mg/mL), Ryutanshakanto (0.625 and 1.25 mg/mL), Tokiinshi (2.5 and 5 mg/mL), Tokito (2.5 and 5 mg/mL), and Saireito (0.625 and 1.25 mg/mL). The six most efficacious Kampo formulations that led to the greatest reduction in sFlt-1 secretion and the lowest sFlt-1/PlGF ratio (Table 1) were progressed for further assessment.

Isolated primary cytotrophoblasts were treated with two concentrations of: Goreisan (5 mg/mL and 10 mg/mL), Tokishakuyakusan (2.5 mg/mL and 5 mg/mL), Rikkunshito (5 mg/mL and 10 mg/mL), Shakuyakukanzoto (1.25 mg/mL and 2.5 mg/mL), Tokiinshi (1.25 mg/mL and 2.5 mg/mL), and Tokito (1.25 mg/mL and 2.5 mg/mL) for 24 h. Based on these findings, the panel was further refined to three Kampo formulations—Goreisan, Tokishakuyakusan, and Shakuyakukanzoto—which were subsequently tested in placental explants (48 h of treatment), isolated primary HUVECs, and commercially sourced UtMVECs. Prior to treatment with Kampo formulations for 24 h, endothelial dysfunction was induced in isolated primary HUVECs and UtMVECs with TNFα (1 ng/mL, Life Technologies) for two hours. All treatments were performed in technical triplicate.

## 3. Elisa

Soluble fms-like tyrosine kinase (sFLT)1 and placental growth factor (PlGF) were measured in conditioned media collected from cytotrophoblast, placental explant, HUVEC, and UtMVEC culture media using the DuoSet Human VEGF R1/Flt-1 (R&D Systems by Bioscience; Minneapolis, MN, USA) and Human PGF ELISA Kit (Invitrogen; Carlsbad, CA, USA), respectively. Optical density for ELISA was measured at 450 nm, determined using a BioRad X-Mark microplate spectrophotometer (BioRad, Hercules, CA, USA), and protein concentrations were calculated using BioRad Microplate Manager 6 software. Media samples were diluted in the following ratios: HUVEC = 1:4; cytotrophoblast = 1:20, and placental explant = 1:20.

### 3.1. Quantitative RT-PCR

Total RNA was extracted from cytotrophoblasts, placental explant, HUVECs, and UtMVECs following treatment using the RNeasy mini kit (Qiagen, Hilden, Germany) and quantified using a Nanodrop ND 1000 spectrophotometer (NanoDrop technologies Inc., Wilmington, NC, USA). RNA was converted to cDNA using the High-Capacity cDNA Reverse Transcription Kit (Applied Biosystems, Waltham, MA, USA) as per manufacturer guidelines. qPCR was performed using Taqman hydrolysis probes for *PlGF* (Hs00182176_m1), *HMOX1* (Hs01110250_m1), *ET-1* (Hs00174961_m1), *IL-1β* (Hs01555410_m1), *NLRP3* (Hs00918082_m1), *VCAM1* (Hs01003372_m1), and *FLT* (Hs01052961_m1) on the CFX 384 (Biorad) using FAM-labelled Taqman universal PCR mastermix (Applied Biosystems) with the following run conditions: 50 °C for 2 min, 95 °C for 10 min, 95 °C for 15 s, 60 °C for 1 min (40 cycles). Cytotrophoblast, HUVEC, and UtMVEC data were normalised to the reference gene, *YWHAZ* (Hs01122454_m1), and placental explant data were normalised to the average of *TOP1* (Hs00243257_m1) and *CYC1* (Hs00357717_m1). Results graphed as fold change relative to control using the 2^−ΔΔCT^ method. The sFlt-1 splice variants *sFlt-1-i13* and *sFlt-1-e15a* were measured with Fast SYBR Green Master mix (Applied Biosystems) using primers specific for each variant as previously published [24], using *YHWAZ* as the reference gene with the following run conditions: 95 °C for 20 s, 95 °C for 1 s, 60 °C for 20 s (40 cycles). All samples were run in technical duplicate.

### 3.2. Statistical Analysis

All in vitro experiments were performed with technical triplicate and repeated greater than or equal to three times using tissue or cells isolated from different patient donors, or passages where cells were commercial. Sample sizes were determined a priori based on power calculations informed by our previous studies using these experimental models [22,23].

Data was assessed for normal distribution prior to statistical analysis. Parametric data were analysed using a one-way ANOVA with Tukey’s multiple comparisons test. Data found to be non-parametric were analysed using the Kruskal–Wallis test, with post hoc analysis with Dunn’s multiple comparison test. Parametric data are presented as either mean ± Standard Error of the Mean (SEM), non-parametric data presented as Median (Range). *p*-values < 0.05 were considered statistically significant. Statistical analyses were performed using GraphPad Prism version 10.0 (GraphPad Software, Boston, MA, USA).

## 4. Results

### 4.1. Kampo Screening in Primary Human Umbilical Vein Endothelial Cells

**Figure 1:**
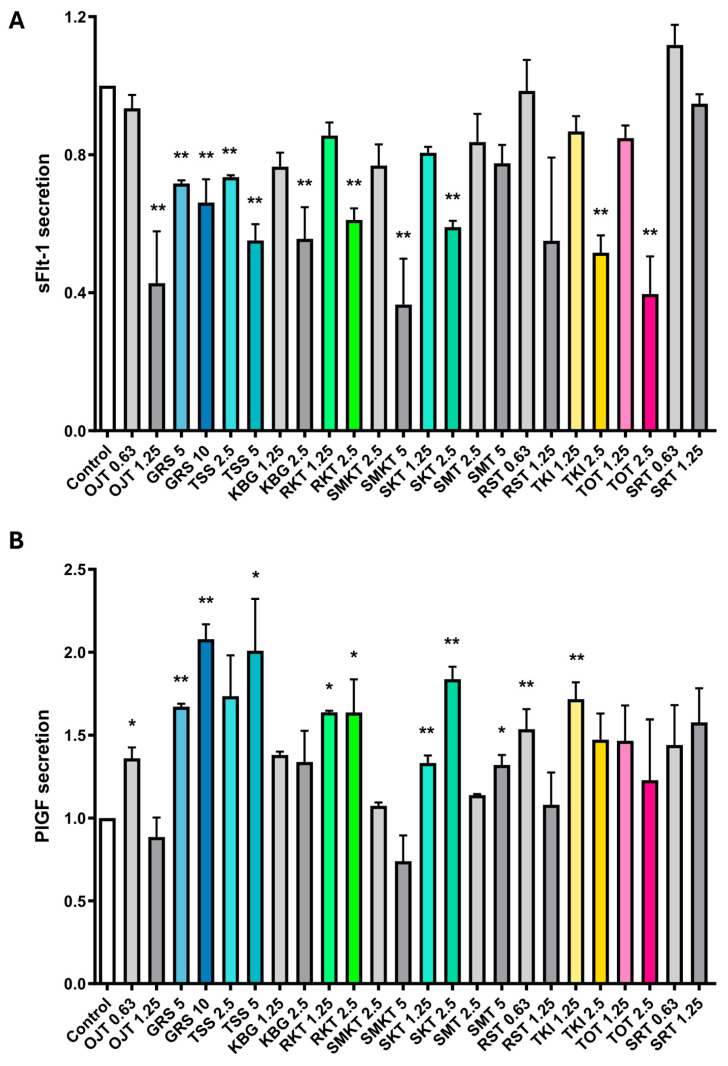
**Kampo screening in human umbilical vein endothelial cells.** Secretion of sFlt-1 (**A**) was significantly decreased with Otsujito 1.25 mg/mL (OJT 1.25), Keishibukuryogan 2.5 mg/mL (KBG 2.5), Rikkunshito 2.5 mg/mL (RKT 2.5), Shichimotsukokato 5 mg/mL (SMKT 5), Shakuyakukanzoto 2.5 mg/mL (SKT 2.5), Tokiinshi 2.5 mg/mL (TKI 2.5), and Tokito 2.5 mg/mL (TOT 2.5), and Goreisan 5 mg/mL (GRS 5) and 10 mg/mL (GRS 10) and Tokishakuyakusan 2.5 mg/mL (TSS 2.5) and 5 mg/mL (TSS 5). Secretion of PlGF (**B**) was significantly increased with Otsujito 0.63 mg/mL (OJT 0.63), Ryutanshakanto 0.63 mg/mL (RST 0.63), and Tokiinshi 1.25 mg/mL (TKI 1.25), TSS 5 and Shimotsuto 5 mg/mL (SMT 5), GRS 5 and GRS 10, Rikkunshito 1.25 mg/mL (RKT 1.25) and RKT 2.5, and Shakuyakukanzoto 1.25 mg/mL (SKT 1.25) and SKT 2.5. Data are expressed relative to control (mean ± SEM; *n* = 3–4). Keishibukuryogan 1.25 mg/mL (KBG 1.5), Shichimotsukokato 2.5 mg/mL (SMKT 2.5), Shimotsuto 2.5 mg/mL (SMT 2.5), Ryutanshakanto 1.25 mg/mL (RST 1.25), Tokito 1.25 mg/mL (TOT 1.25), Saireito 0.63 mg/mL (SRT 0.63) and 1.25 mg/mL (SRT 1.25), * *p* < 0.05, ** *p* < 0.01.

We initially screened 12 Kampo formulations to determine their effect on sFlt-1 and PlGF secretion from primary HUVECs. Secretion of sFlt-1 was significantly reduced (compared to control vehicle) with high-dose Otsujito, Keishibukuryogan, Rikkunshito, Shichimotsukokato, Shakuyakukanzoto, Tokiinshi, and Tokito treatment. Both low and high-dose Goreisan and Tokishakuyakusan also reduce sFlt-1 secretion compared to control; however, sFlt-1 secretion was unaffected by Shimotsuto, Ryutanshakanto, and Saireito (Figure 1A). Secretion of PlGF was significantly increased with low-dose Otsujito, Ryutanshakanto, and Tokiinshi, high-dose Tokishakuyakusan and Shimotsuto, low- and high-dose Goreisan, Rikkunshito, and Shakuyakukanzoto, and unaffected by Keishibukuryogan, Shichimotsukokato, Tokito, or Saireito (Figure 1B).

The clinically relevant sFlt-1/PlGF ratio was calculated using the higher Kampo dose and is presented in Table 1. Goreisan (0.32), Tokishakuyakusan (0.27), Rikkunshito (0.37), Shakuyakukanzoto (0.32), Tokiinshi (0.35), and Tokito (0.32) had the lowest ratios and were selected for further functional experiments.

### 4.2. Kampo Treatment of Isolated Primary Human Trophoblast Cells

**Figure 2:**
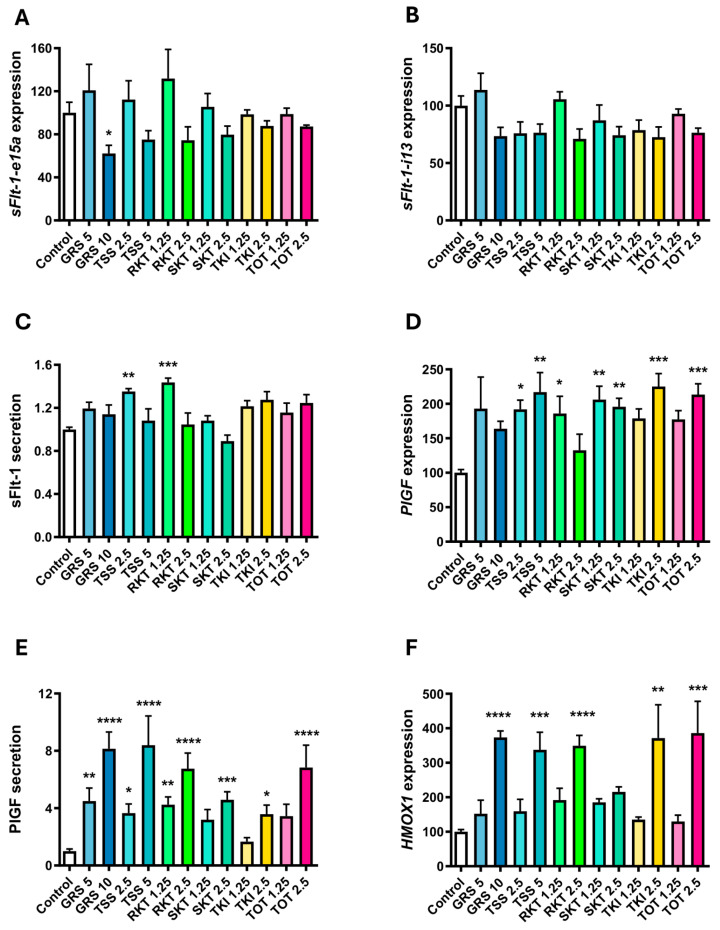
Isolated primary trophoblast expression of *sFlt-1* isoforms *sFlt-1-e15a* (**A**) and *sFlt-1-i13* (**B**) was not altered by Kampo treatment, except Goreisan 10 mg/mL (GRS 10), which significantly decreased *sFlt-1-e15a* expression. Secretion of sFlt-1 (**C**) was significantly increased with Tokishakuyakusan 2.5 mg/mL (TSS 2.5) and Rikkunshito 1.25 mg/mL (RKT 1.25), but was unaffected by all other Kampo treatments. Expression (**D**) and secretion (**E**) of PlGF was significantly increased with Goreisan 5 mg/mL (GRS 5, secretion only) and GRS 10 (secretion only), TSS 2.5 and Tokishakuyakusan 5 mg/mL (TSS 5), RKT 1.25 and Rikkunshito 2.5 mg/mL (RKT 2.5, secretion only), Shakuyakukanzoto 1.25 mg/mL (SKT 1.25, expression only) and 2.5 mg/mL (SKT 2.5), Tokiinshi 2.5 mg/mL (TKI 2.5), and Tokito 2.5 mg/mL (TOT 2.5). Expression of antioxidant *HMOX1* (**F**) was significantly increased with GRS 10, TSS 5, RKT 2.5, TKI 2.5, and TOT 2.5. Data are mean ± SEM, expressed relative to control. *n* = 3–5 trophoblast isolations from different placentas. Tokiinshi 1.25 mg/mL (TKI 1.25), Tokito 1.25 mg/mL (TOT 1.25), * *p* < 0.05, ** *p* < 0.01, *** *p* < 0.001, **** *p* < 0.0001.

Except for high-dose Goreisan, which significantly reduced *sFlt-1-e15a* expression, Kampo treatment had no effect on *sFlt-1-e15a* and *sFlt-1-i13* expression in isolated primary human trophoblasts (Figure 2A and Figure 2B, respectively). Low-dose Tokishakuyakusan and low-dose Rikkunshito significantly increased total sFlt-1 protein secretion from trophoblast, while other Kampo treatments had no effect (Figure 2C). Trophoblast PlGF expression (Figure 2D) and secretion (Figure 2E) significantly increased with low and high dose Goreisan (secretion only), Tokishakuyakusan, Rikkunshito, Shakuyakukanzoto, as well as high dose Tokiinshi, and Tokito. Except for Shakuyakukanzoto, the high dose of each Kampo significantly increased trophoblast *HMOX1* expression (Figure 2F). Kampo treatment did not negatively affect the viability of isolated primary human trophoblast cells (Supplementary Figure S1A).

### 4.3. Kampo Treatment of Primary Human Placental Explants

**Figure 3:**
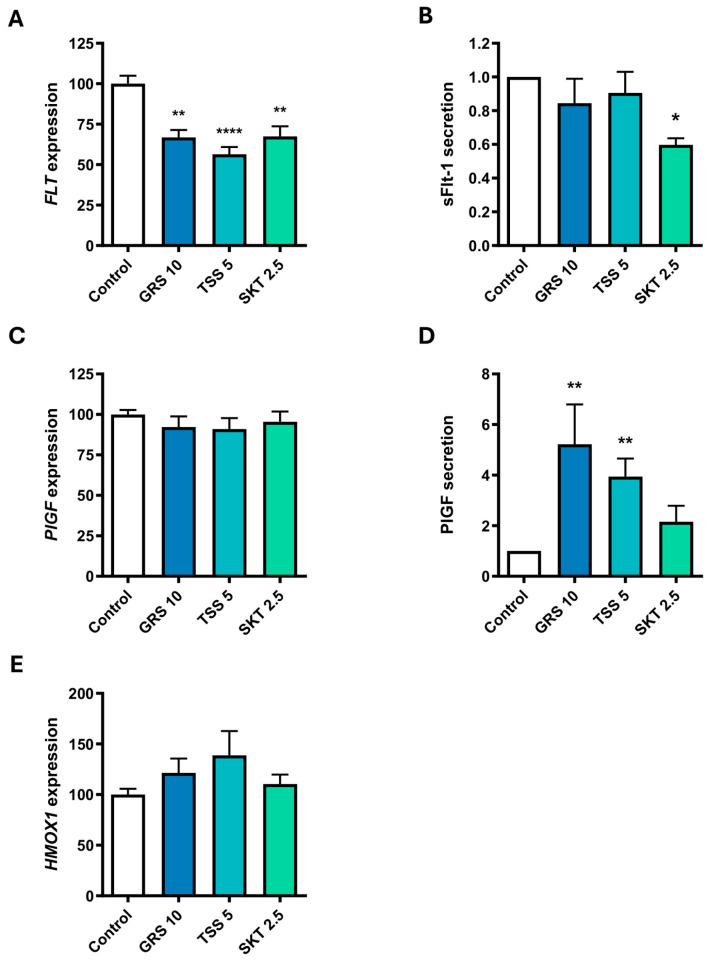
Placental explant expression of *FLT* (**A**) was significantly decreased with Goreisan 10 mg/mL (GRS 10), Tokishakuyakusan 5 mg/mL (TSS 5), and Shakuyakukanzoto 2.5 mg/mL (SKT 2.5). Secretion of sFlt-1 (**B**) was significantly reduced with SKT 2.5. Expression of *PlGF* (**C**) and *HMOX1* (**E**) was unaffected by Kampo treatment; however, secretion of PlGF (**D**) was significantly increased with GRS 10 and TSS 5. Data are mean ± SEM, expressed relative to control. *n* = 4–5 placentas. * *p* < 0.05, ** *p* < 0.01, **** *p* < 0.0001.

Goreisan, Tokishakuyakusan, and Shakuyakukanzoto significantly reduced total *FLT* expression in placental explants (Figure 3A); however, only Shakuyakukanzoto decreased sFlt-1 protein secretion (Figure 3B). Kampo treatment did not alter *PlGF* expression in placental explants (Figure 3C); however, Goreisan and Tokishakuyakusan significantly increased PlGF secretion (Figure 3D). None of the Kampo treatments altered placental explant expression of *HMOX1* (Figure 3E), a key antioxidant cytoprotective molecule.

### 4.4. Kampo Treatment of Isolated Primary Human Umbilical Vein Endothelial Cells Following TNFα-Induced Endothelial Dysfunction

**Figure 4:**
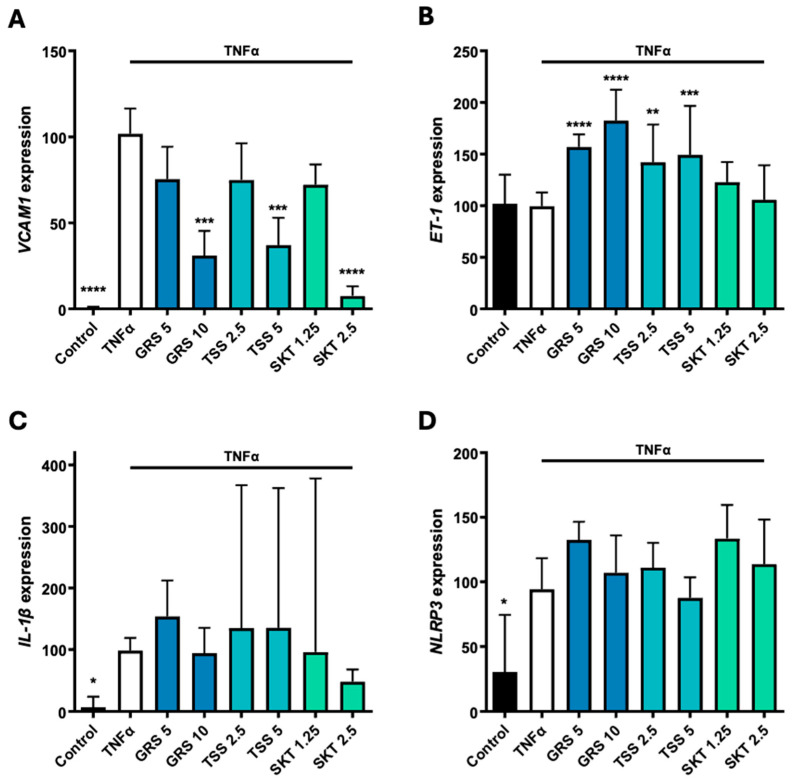
Isolated primary HUVECs with TNFα treatment show a significant increase in expression of *VCAM1* (**A**); this increase was decreased with Goreisan 10 mg/mL (GRS 10), Tokishakuyakusan 5 mg/mL (TSS 5), and Shakuyakukanzoto 2.5 mg/mL (SKT 2.5). Expression of *ET-1* (**B**) was unaffected by TNFα but was significantly increased with Goreisan 5 mg/mL (GRS 5) and GRS 10, and Tokishakuyakusan 2.5 mg/mL (TSS 2.5) and TSS 5. Expression of *IL-1β* (**C**) and *NLRP3* (**D**) was both increased with TNFα and unaffected by Kampo treatment. Data are median (range), expressed relative to TNFα-treated controls. *n* = 3 HUVEC isolations from different umbilical cords. Shakuyakukanzoto 1.25 mg/mL (SKT 1.25), * *p* < 0.05, ** *p* < 0.01, *** *p* < 0.001, **** *p* < 0.0001; compared to TNFα control.

TNFα significantly increased *VCAM1*, *IL-1β*, and *NLRP3* expression in HUVECs but had no effect on *ET-1* (Figure 4). High-dose Goreisan, Tokishakuyakusan, and Shakuyakukanzoto significantly reduced *VCAM1* expression (Figure 4A). Goreisan and Tokishakuyakusan significantly increased *ET-1* expression in HUVECs (Figure 4B). Kampo treatment did not alter *IL-1β* (Figure 4C) or its upstream activator *NLRP3* (Figure 4D). Kampo treatment did not alter the viability of isolated primary HUVECs (Supplementary Figure S1B).

### 4.5. Kampo Treatment of Uterine Microvascular Endothelial Cells Following TNFα-Induced Endothelial Dysfunction

**Figure 5:**
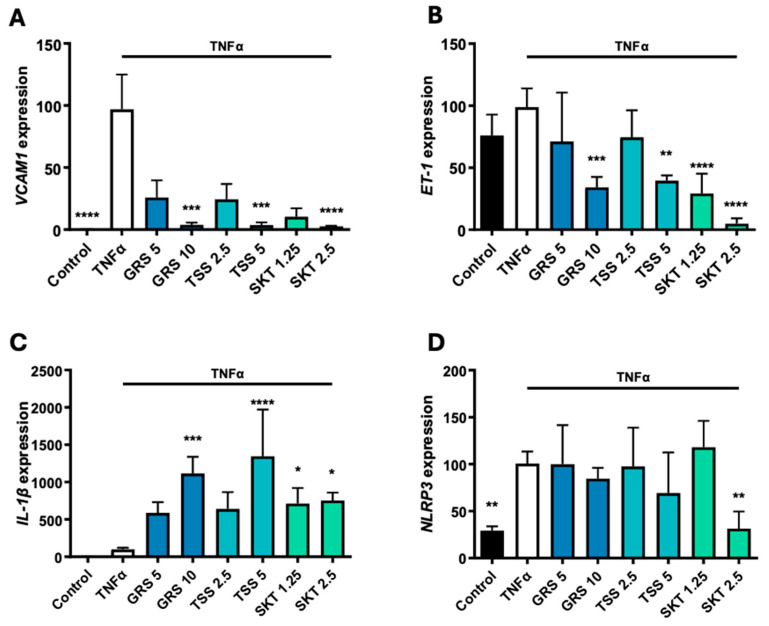
Isolated UtMVEC treated with TNFα show a significant increase in expression of *VCAM1* (**A**), which was subsequently decreased with Goreisan 10 mg/mL (GRS 10), Tokishakuyakusan 5 mg/mL (TSS 5), and Shakuyakukanzoto 2.5 mg/mL (SKT 2.5). Expression of *ET-1* (**B**) was unaffected by TNFα but was significantly decreased with GRS 10, TSS 5, Shakuyakukanzoto 1.25 mg/mL (SKT 1.25) and 2.5 mg/mL (SKT 2.5). Expression of *IL-1β* (**C**) was unaffected by TNFα but was significantly increased with GRS 10, TSS 5, and SKT 1.25 and SKT 2.5. TNFα significantly increased expression of *NLRP3* (**D**), which was subsequently decreased by SKT 2.5. Data are median (range), expressed relative to TNFα. *n* = 3 experimental replicates. Goreisan 5 mg/mL (GRS 5), Tokishakuyakusan 2.5 mg/mL (TSS 2.5), * *p* < 0.05, ** *p* < 0.01, *** *p* < 0.001, **** *p* < 0.0001.

As with the HUVEC experiment, TNFα treatment caused a significant increase in *VCAM1* and *NLRP3* expression in UtMVECs, but in contrast, had no effect on *ET-1* or *IL-1β* (Figure 5). At the higher dose, Goreisan, Tokishakuyakusan, and Shakuyakukanzoto treatment significantly reduced endothelial dysfunction markers *VCAM1* and *ET-1* mRNA expression (Figure 5A and Figure 5B, respectively). Conversely, high-dose Goreisan, Tokishakuyakusan, and Shakuyakukanzoto significantly increased *IL-1β* expression (Figure 5C). Shakuyakukanzoto treatment caused a significant decrease in *NLRP3* expression (Figure 5D). Low and high dose Goreisan and high dose Tokishakuyakusan enhanced UtMVEC viability, whereas Shakuyakukanzoto had no significant effect (Supplementary Figure S1C).

The data summarised in Table 2 show that Goreisan and Tokishakuyakusan exhibited the highest number of significant beneficial changes among the Kampo formulations tested. While this is a somewhat simplistic subjective assessment, we provide the spectrum of effects of the Kampo formulation for the interpretation of the findings.

## 5. Discussion

In this study, we investigated the therapeutic potential of several Kampo formulations (Japanese traditional herbal medicines) in pathological models of preeclampsia. Our findings indicate that select Kampo formulations can modulate angiogenic factors, oxidative stress markers, and endothelial dysfunction in primary human cells and tissues from pregnancy. Goreisan, Tokishakuyakusan, Rikkunshito, Shakuyakukanzoto, Tokiinshi, and Tokito increased pro-angiogenic PlGF in isolated placental trophoblast cells and tissue and might be beneficial in restoring angiogenic balance in preeclampsia. Notably, Tokishakuyakusan and Rikkunshito induced modest increases in anti-angiogenic sFlt-1 in isolated trophoblast cells. However, this effect was not observed in placental explants, which contain multiple placental cell types and intact tissue architecture. In these more physiologically complex tissues, Goreisan, Tokishakuyakusan, and Shakuyakukanzoto instead decreased total *FLT* mRNA expression (and sFlt-1 secretion with Shakuyakukanzoto). By increasing PlGF and decreasing sFlt-1, these Kampo formulations shift the angiogenic environment towards a more pro-angiogenic state, which may help alleviate endothelial dysfunction and mitigate the development of preeclampsia [25].

Additionally, these Kampo formulations (except Shakuyakukanzoto) significantly upregulated *HMOX1* expression in isolated trophoblast cells, a key regulator of antioxidant defence, pointing to a key cytoprotective role against oxidative stress. In endothelial cells, Goreisan, Tokishakuyakusan, and Shakuyakukanzoto significantly reduced the expression of *VCAM1*, a key adhesion molecule that both marks and contributes to endothelial dysfunction and inflammatory cell recruitment [26], with implications in preeclampsia [27]. This suggests a potential vascular-protective effect that could help preserve endothelial integrity during pregnancy. However, it is important to note that the direct effects these Kampo had on endothelial cells varied compared to other cell models used. In HUVECs, Kampo treatments increased *ET-1* and had no effect on *IL-1β* or *NLRP3*. In UtMVECs, Kampo treatments decreased *ET-1* but increased IL-1β without affecting *NLRP3*. Thus, these divergent effects on *ET-1* may reflect important differences in the functional characteristics of the two endothelial cell models, with HUVECs potentially being more responsive to pro-vasoconstrictor stimuli, while UtMVECs may be more sensitive to the regulatory effects of Kampo on vascular tone and inflammation [28,29]. *ET-1* is a potent vasoconstrictor that contributes to increased vascular resistance and elevated blood pressure, key features of preeclampsia, making its reduction an important therapeutic goal [30].

While the Kampo formulations we investigated did not significantly reduce markers of inflammation, *IL-1β,* and *NLRP3*, there are numerous other Kampo preparations that could be explored for their potential anti-inflammatory properties in isolated cells and tissues from pregnancy [31]. Thus, further investigation is warranted, potentially across a broader dose range, to better understand the preclinical pharmacological aspects of Kampo in gestational tissues.

Previous studies, particularly on Tokishakuyakusan, have demonstrated its efficacy in a model of preeclampsia [32,33]. In Japanese clinical practice, it has been used for mild cases of HDP and pregnancy-related abnormalities such as recurrent pregnancy loss; however, there is a lack of evidence based on controlled clinical trials. Recent basic research has shown its effects on PlGF elevation [34] and immune modulation of invariant-natural killer T cells [35]; in this study, we showed its modulatory effects on angiogenic factors and antioxidant cytoprotective molecules in human primary endothelial cells or trophoblast cells/tissues.

Overall, our data highlights the therapeutic potential of select Kampo formulations, specifically for mediating key mechanisms implicated in the pathogenesis of preeclampsia, including improving the balance in angiogenic/anti-angiogenic secretion, enhanced antioxidant/cytoprotection, and endothelial dysfunction. Outside of pregnancy, Goreisan has been shown to protect against oxidative stress-induced cardiac remodelling [36], while Rikkunshito has demonstrated protective effects against atrial fibrosis [37] and inflammation-induced renal injury [38]. These protective mechanisms may be particularly beneficial in preeclampsia, where oxidative stress, endothelial dysfunction, and organ damage contribute to the condition’s pathogenesis. Additionally, such effects could help mitigate the long-term cardiovascular risk often seen in individuals post-preeclampsia [39,40,41,42,43,44]. These properties suggest that Kampo formulations could offer a multi-targeted approach to managing not only preeclampsia but also its long-term complications, such as cardiovascular disease, making them a promising therapeutic avenue for improving maternal health outcomes; however, further clinical studies would be needed to determine this.

In vitro studies are highly effective for screening beneficial drugs or compounds from multiple chemical substances. However, a limitation of our study is that, unlike Western medicine, which typically consists of a single active ingredient, Kampo medicine is a complex mixture containing a vast number of compounds. It is also important to note that this is a limitation of the current study; further studies evaluating each bioactive compound within the Kampo formulation would be necessary to elucidate precise mechanisms. The mechanistic actions underlying the Kampo effects are highly intricate, making it extremely challenging to functionally analyse each active ingredient and elucidate their interactions. Primary cytotrophoblasts, endothelial cells, and placental explants offer important in vivo and ex vivo findings relative to specific aspects of preeclampsia, but they cannot reproduce the complex maternal–fetal interactions that would occur in vivo. Importantly, these models do not fully recapitulate the specific metabolic, systemic inflammation, immune responses, or hemodynamic changes that occur in vivo. Further studies in appropriate animal models, together with pharmacokinetic and safety evaluations, will be necessary to determine the translational potential of these formulations for the prevention or treatment of preeclampsia. Furthermore, while a strength of the current study is the first evaluation in primary human gestational tissues, it is important to note the limited sample size utilised.

Additionally, some components of Kampo medicine are known to undergo activation or deactivation through metabolic processes involving the liver and intestinal microbiota. Other factors, such as absorption, distribution, and elimination, can also influence the concentration of Kampo ingredients in local tissues. Therefore, findings from in vitro studies do not necessarily reflect their effects in vivo, and the determination of physiological relevance needs further research. Nevertheless, it is intriguing that Tokishakuyakusan and Goreisan, which have been clinically recognised as beneficial for hypertensive disorders in pregnancy, were found to exert favourable effects on the pathophysiological markers of preeclampsia. These effects include the promotion of angiogenic factors such as PlGF in trophoblast cells and HUVECs, as well as the upregulation of antioxidant molecules like HMOX1.

In conclusion, our data provides the first exploration of selected Kampo herbal formulations to specifically modulate key pathways implicated in preeclampsia pathophysiology, including angiogenic imbalance, oxidative stress, and endothelial dysfunction in primary human preclinical models. Given their multi-target pharmacological profile and growing use in pregnancy [20,21,45,46,47], these findings support further investigation of Kampo medicines as potential novel therapeutic candidates for preeclampsia. Future studies, in appropriate in vivo preclinical models and clinical settings, are needed to further determine their efficacy, safety [48] and mechanisms of action.

## Figures and Tables

**Table 1:** sFlt-1 and PlGF secretion from HUVECs.

Kampo		sFlt-1 (%) *	PlGF (%) *	sFlt-1/PlGF
Otsujito	OJT	42.7	88.67	0.48
Goreisan	GRS	66.1	207.9	0.32
Tokishakuyakusan	TSS	55.2	200.9	0.27
Keishibukuryogan	KBG	55.6	133.9	0.42
Rikkunshito	RKT	61.1	163.6	0.37
Shichimotsukokato	SMKT	36.6	73.9	0.49
Shakuyakukanzoto	SKT	59.0	183.6	0.32
Shimotsuto	SMT	77.5	132.1	0.59
Ryutanshakanto	RST	55.0	108.0	0.51
Tokiinshi	TKI	51.6	147.3	0.35
Tokito	TOT	39.6	122.8	0.32
Saireito	SRT	94.8	157.8	0.60

* Data expressed as a % of secretion relative to control, *n* = 3–4.

**Table 2:** Summary of the data investigated with Kampo formulations and preeclampsia indices.

Cells Tissues	Indices	GRS	TSS	RKT	SKT	TKI	TOT
**Primary HUVECs**	sFlt-1 secretion	**↓**	**↓**	**↓**	**↓**	**↓**	**↓**
	PlGF secretion	**↑**	**↑**	**↑**	**↑**	**↑**	**→**
**Primary Trophoblasts**	*e15A* expression	**↓**	**→**	**→**	**→**	**→**	**→**
	*i13* expression	**→**	**→**	**→**	**→**	**→**	**→**
	sFlt-1 secretion	**→**	**↑**	**↑**	**→**	**→**	**→**
	*PlGF* expression	**→**	**↑**	**↑**	**↑**	**↑**	**↑**
	PlGF secretion	**↑**	**↑**	**↑**	**↑**	**↑**	**↑**
	*HMOX1* expression	**↑**	**↑**	**↑**	**→**	**↑**	**↑**
	Viability	**→**	**→**	**→**	**→**	**→**	**→**
**Placental Explants**	*FLT* expression	**↓**	**↓**	**-**	**↓**	**-**	**-**
	sFlt-1 secretion	**→**	**→**	**-**	**↓**	**-**	**-**
	*PlGF* expression	**→**	**→**	**-**	**→**	**-**	**-**
	PlGF secretion	**↑**	**↑**	**-**	**→**	**-**	**-**
	*HMOX1* expression	**→**	**→**	**-**	**→**	**-**	**-**
**TNF** **α** **-stimulated HUVECs**	*VCAM1* expression	**↓**	**↓**	**-**	**↓**	**-**	**-**
	*ET-1* expression	**↑**	**↑**	**-**	**→**	**-**	**-**
	*IL-1β* expression	**→**	**→**	**-**	**→**	**-**	**-**
	*NLRP3* expression	**→**	**→**	**-**	**→**	**-**	**-**
	Viability	**→**	**→**	**-**	**→**	**-**	**-**
**TNF** **α** **-stimulated** **UtMVECs**	*VCAM1* mRNA	**↓**	**↓**	**-**	**↓**	**-**	**-**
	*ET-1* mRNA	**↓**	**↓**	**-**	**↓**	**-**	**-**
	*IL-1β* mRNA	**↑**	**↑**	**-**	**↑**	**-**	**-**
	*NLRP3* mRNA	**→**	**→**	**-**	**↓**	**-**	**-**
	Viability	**↑**	**↑**	**-**	**→**	**-**	**-**
**Number of favourable changes**		**11**	**11**	**5**	**10**	**5**	**4**
**Number of unfavourable changes**		**2**	**3**	**1**	**1**	**0**	**0**

Goreisan (GRS), Tokishakuyakusan (TSS), Rikkunshito (RKT), Shakuyakukanzoto (SKT), Tokiinshi (TKI), Tokito (TOT). Blue arrows depict favourable effects with treatment in preeclampsia pathology. Red arrows depict unfavourable effects of treatment in preeclampsia pathology.

## Data Availability

Data available upon reasonable request.

## Supplementary Materials

The following supporting information can be downloaded at https://www.mdpi.com/article/10.3390/antiox15070877/s1, Supplementary Figure S1: Cellular viability following treatment with Kampo. None of the Kampo formulations affected the viability of isolated primary cytotrophoblast cells (A) or human umbilical vein endothelial cells (B). Goreisan 5 mg/mL (GRS 5) and 10 mg/mL (GRS 10), and Tokishakuyakusan 5 mg/mL (TSS 5) significantly increased uterine microvascular endothelial cell viability following TNFα-induced endothelial dysfunction (C). Data are mean ± SEM, expressed relative to control (A) or TNFα (B and C). *n* = 3 experimental replicates. Tokishakuyakusan 2.5 mg/mL (TSS 2.5), and Shakuyakukanzoto 1.25 mg/mL (SKT 1.25) and 2.5 mg/mL (SKT 2.5), * *p* < 0.05. *** = *p* < 0.001.
