# Supplementary material for: Kampo Medicines Modulate Angiogenic, Antioxidant, and Inflammatory Pathways in Human Preclinical Models: Implications for Preeclampsia
Source: Antioxidants (Basel). 2026 Jul 14;15(7):877. doi: 10.3390/antiox15070877 (PMC13403422; doi:10.3390/antiox15070877)

Supplementary Figure S1. Cellular viability following treatment with Kampo. None of the Kampo formulations affected the viability of isolated primary cytotrophoblast cells (A), or human umbilical vein endothelial cells (B). Goreisan 5 mg/ml (GRS 5) and 10 mg/ml (GRS 10), and Tokishakuyakusan 5 mg/ml (TSS 5) significantly increased uterine microvascular endothelial cell viability following TNFα-induced endothelial dysfunction (C). Data are mean ± SEM, expressed relative to control (A) or TNFα (B and C). n=3 experimental replicates. Tokishakuyakusan 2.5 mg/ml (TSS 2.5), and Shakuyakukanzoto 1.25 mg/ml (SKT 1.25) and 2.5 mg/ml (SKT 2.5), *p<0.05. *** = p < 0.001.

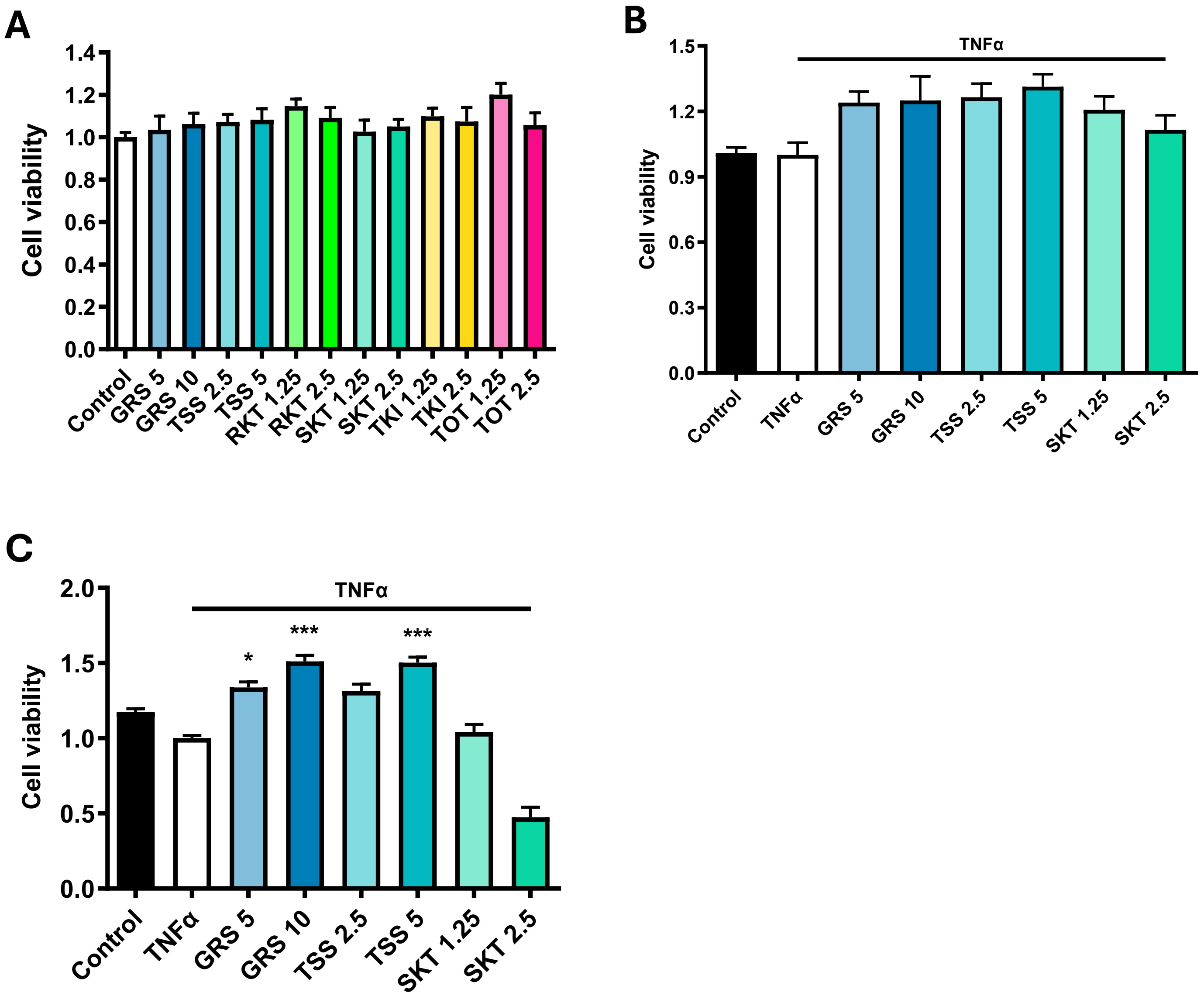

Supplement: Supplementary file 1 [file antioxidants-15-00877-s001.zip › antioxidants-4394789-supplementary.pdf]
